# Preview Control of a Semi-Active Suspension System Supplemented by an Active Aerodynamic Surface

**DOI:** 10.3390/s25226922

**Published:** 2025-11-12

**Authors:** Syed Babar Abbas, Iljoong Youn

**Affiliations:** Department of Mechanical and Aerospace Engineering, Gyeongsang National University, 501, Jinju-daero, Jinju-si 52828, Gyeongsangnam-do, Republic of Korea; engr.babar647@gnu.ac.kr

**Keywords:** passengerride comfort, optimal preview control, aerodynamic control surface, semi-active suspension system

## Abstract

This research presents a harmonized optimal preview control strategy for a semi-active suspension system (SASS) with a controlled damper varied between the upper and lower bounds of the damping coefficient and an active aerodynamic surface (AAS) control. The preview control algorithm is based on a simplified bilinear 2-DOF quarter-car model to address the tradeoff between passenger ride comfort and road holding capabilities. While the active suspension with the actuator requires a significant amount of energy to provide control force, the semi-active suspension system with a variable damping coefficient mechanism consumes minimal energy to adapt quickly to the real-time operating conditions. Moreover, the dynamic performance of semi-active suspension with the preview controller in conjunction with the active aerodynamic surface is significantly improved. MATLAB^®^ (R2025b)-based numerical simulations for different road excitations were carried out for the evaluation of the proposed system. Both time-domain and frequency-domain results demonstrate enhanced vehicle dynamic performances in response to road bumps, asphalt road excitations, and harmonic input signals. The simulation performance results indicate that the proposed system extraordinarily reduced the variation in the mean-squared value of the car body vertical acceleration. At the same time, the system enhanced the wheel-road holding metric by decreasing the variation in the gripping force on the ground surface, while maintaining the necessary suspension rattle space constraints within the prescribed limit.

## 1. Introduction

### 1.1. Background

A vehicle suspension system plays a crucial role in enhancing ride comfort, handling stability, and passenger safety. It is responsible for absorbing external shocks from the road irregularities and maintaining tire contact on the road surface. At the same time, the suspension system provides structural support during various road maneuvers. Depending on the level of control, suspension systems are classified as passive, semi-active, and fully active [1]. A passive suspension system (PSS) is easy to implement, and it has been used continuously in the past. It consists of a fixed spring and damper mechanism and has limited handling capability. However, it cannot offer robust and reliable performance under extreme road and speed conditions [2]. Active suspension system (ASS) improves the vehicle’s driving safety and handling performance by utilizing active actuator force to attenuate dynamic road excitations. Meanwhile, the SASS consumes a small amount of external power to vary the variable damper’s orifice and change the amount of current in the MR damper if the condition for energy dissipation is fulfilled [3].

### 1.2. Related Work

So far, many studies have focused on addressing the tradeoff between the conflicting objectives of driving comfort and ground holding by employing various suspension configurations and control methodologies [4]. A nonlinear quarter-car and half-car model with a stochastic noise signal using method of spectral decomposition was employed to obtain the desired responses. The control force, suspension acceleration, and road holding metrics were calculated using preview information. Various velocities were considered for the simulation. However, the performance improved for some preview distances, beyond which the saturation occurred [5,6]. A high-order incremental model predictive controller (MPC) scheme incorporating additional information was proposed based on AAS for the lead vehicle (quarter-car model). The preview sensors estimated the road profile. The robust stability analysis was compared with the conventional MPC system. The proposed method outperforms other control suspensions by utilizing preview information. However, the work did not consider the model uncertainty analysis in the formulated system [7].

To address the problem associated with unsprung mass and improve the vertical performance, a preview-based collaborative control method (MPC) was formulated for a knee-wheeled legged robot (KWLR). The wheel-legged system is approximated by a quarter of the vehicle system. The combined MPC and PI controllers’ output was provided as input to the preview controller. The results demonstrate that the magnitude of the sprung mass vertical acceleration of the KWLR is reduced. However, the effect of unknown road excitation signals on different roads were not considered [8]. An MPC-based nonlinear SASS has been demonstrated to optimize the suspension performance for the full car dynamic model to compensate for the shift delay and to improve the ride performance. All the states were estimated by using the Kalman filter. The road preview information was obtained through temporal–spatial conversion and relative car body motion for a specific time using a built-in camera. However, it has been observed that the rear suspension velocity is not accurate owing to the model uncertainties in the scanned information [9].

A numerical simulation study was performed to enhance the driving comfort of a half-car model utilizing vehicle-to-vehicle (V2V) communication for the ego vehicle. The ASS employed optimal preview information to track the wheel motion (disturbances), which was received from the preceding vehicle using radar or light detection and ranging (LiDAR) signals. Some of the issues of the look-ahead sensor were addressed. However, the results were compared only with the feedback controller, and the dynamic stability analyses were not considered [10]. In another study, the author employed a novel hybrid varying (HV-MPC) optimization method to enhance the passenger comfort and speed planning of a SASS over a fixed length of road preview elevation. The control scheme transforms the processed data from the spatial domain to adapt to variations in the speed of autonomous vehicles (AVs) using dynamic programming. The combined results were tested to optimize vertical acceleration, trajectory planning, and driving time. Meanwhile, road grasping of the AV was not considered, and vertical acceleration was ignored during speed planning [11]. A sliding mode controller (SMC) was employed to improve the driving comfort and road holding metric of a hybrid SASS using a quarter-of-vehicle (QoV) model. The conformance was performed using a functional mock-up interface (FMI). A convex set was selected for three cases of comfort and holding attributes to support the results. However, this study does not consider the impact on the suspension stroke, ground profile, and speed limitations of the vehicle suspension [12].

To mitigate the anticipated road disturbances and enhance dynamic vehicle comfort, a robust discrete-time, preview-based controller was designed for the automotive suspension system. The multimodel control algorithm of SASS using an electrorheological (ER) damper enhanced the performance of the system using feature prediction of the road profile in real time for a 1/5-car test bench. The numerical simulation results were evaluated for vertical comfort using the feedback–feedforward (FBFF) control approach and the feedback (FB) approach for both the time and frequency domains. Nonetheless, the study did not take into account the rattle space requirement and dynamic tire holding metrics [13].

There has been renewed interest in improving the vertical performance of a vehicle using a movable active aerodynamic control surface (AACS) for a sports car. The author in [14] employed an integrated control approach by using two AACS and differential braking control (DBC) system to enhance dynamic stability during high-speed maneuvers. For the desired yaw moment, SMC was designed for the upper control surface, and an optimal controller was employed for coordination and dynamic brake pressure control. The numerical simulation revealed that the proposed control strategy can effectively improve vehicle dynamic stability and wheel workload usage. However, this study did not address the important metrics of passenger comfort and suspension travel. The author in [15] utilized an AAS for the attitude control of a half-car model using a passive suspension system. The predictive control strategy successfully mitigated the amplitude of vibration and improved the chassis’ vertical acceleration and dynamic tire grip on the road surface. The study utilized the preview sensor information ahead of the vehicle’s forward motion before enabling an anticipative control action to be taken for the isolation of external disturbances. In a related study, a control flap mechanism was implemented for the rear wing of a sports car to enhance aerodynamic performance through CAD analysis. The results show that a downward force can be generated, which enhances the maneuverability and versatility of the vehicle for different spoiler configurations [16]. A review paper highlighted various controller schemes and strategies that were developed to enhance vehicle handling, ride comfort, and safety [17]. The paper also highlighted several issues faced by the suspension system, which means that there is still a lack of knowledge of various robust algorithms and strategies for commercial vehicles.

### 1.3. Objectives

In light of the above research findings, this study focuses on the design and synthesis of an efficient optimal preview controller. By using look-ahead preview sensors, the proposed controller anticipates the road disturbances in advance, thereby enhancing the target performance indices. The model is augmented by an active aerodynamic surface that varies the vertical load distribution of the sprung mass in response to the externally induced road excitations. To preserve the integrity of the suspension system, the model maintains the necessary suspension rattle space requirement within the nominal limit. The objectives of this work can be summarized briefly as follows:

1. To synthesize and simulate the state-space model of a semi-active suspension system combined with an active aerodynamic control surface to enhance passenger ride comfort and the total performance criterion without compromising on the road holding.
2. To design an optimal preview control system in conjunction with an AAS that comprises feedback and feedforward parts to take anticipatory action ahead of time, thereby minimizing the effect of external disturbances on the suspension system.
3. To evaluate the performance of the proposed model by comparing it with other suspensions and to carry out a robust comparative analysis of the target suspension metrics in both the time and frequency domains.
4. To perform computational analyses while keeping the suspension travel requirements and the passivity constraints of the SASS within the prescribed limits.

The rest of this paper is organized as follows: Section 2 presents a mathematical model of a semi-active suspension system combined with an active aerodynamic surface. Problem formulation is illustrated in Section 3. Section 4 outlines the preview control design strategy. Section 5 presents the results and discussions. Finally, the study is concluded in Section 6.

## 2. Vehicle Analysis Model

### 2.1. Quarter Car with Semi-Active Suspension

**Figure 1:**
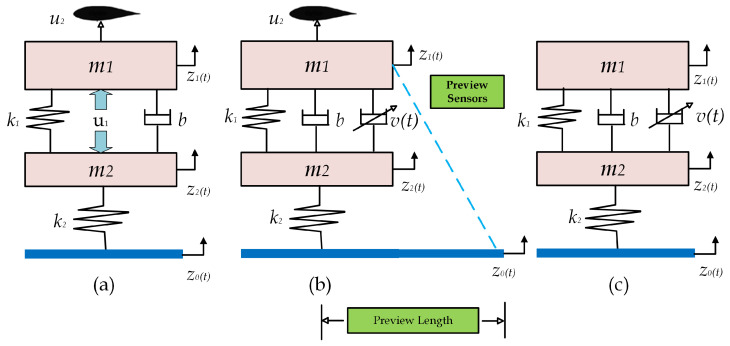
Different suspensions of 2-DOF quarter-of-vehicle model: (**a**) ASS with an active airfoil [18], (**b**) SASS with an active airfoil and preview part, (**c**) SASS only.

The governing equations of a simplified quarter-car model for the ASS and nonlinear SASS are shown in Figure 1. This QoV model is used widely in the literature, and incorporates all the essential characteristics of the detailed model. The model is augmented by an active aerodynamic control surface and preview sensors that contain anticipated future information about a road’s uneven conditions.

Equations (1) and (2) are derived using Newton’s equation of motion about the static equilibrium point in the vertical direction.

(1) $$\begin{array}{cc} m_{1}{\ddot{z}}_{1}+k_{1}\left(z_{1}-z_{2}\right)+\left[b+v\left(t\right)\right]\left({\dot{z}}_{1}-{\dot{z}}_{2}\right)-u_{2} & =0 \end{array}$$

(2) $$\begin{array}{cc} m_{2}{\ddot{z}}_{2}+k_{1}\left(z_{2}-z_{1}\right)+\left[b+v\left(t\right)\right]\left({\dot{z}}_{2}-{\dot{z}}_{1}\right)+k_{2}\left(z_{2}-z_{o}\right) & =0 \end{array}$$

where v(t) is the variable damper coefficient. Table 1 presents the details of the model parameters used in the simulation. The sprung mass $m_{1}$ depicts a quarter of the vehicle’s chassis, internal components, and frame, $m_{2}$ corresponds to the wheel suspension, and multiple-link assembly from the vehicle chassis to the ground surface. The passive linear components of the spring and damper coefficient are represented by $k_{1}$ and *b*, while the tire compressibility is denoted by $k_{2}$. The variable $z_{0}$ represents the vertical road disturbance profile. The vertical absolute displacements of the sprung mass and wheel bounce are denoted by $z_{1}$, $z_{2}$, respectively. For the performance analysis, the tire damping is neglected as its effect is negligible compared to the wheel stiffness.

The time-varying excitation velocity w(t) enters through the wheel assembly, impacting passenger comfort and safety. To develop the state space model of the vehicle, the state vector is defined as

(3) $$x={\left[z_{1}-z_{2},\;{\dot{z}}_{1},\;z_{2}-z_{0},\;{\dot{z}}_{2}\right]}^{T},w\left(t\right)={\dot{z}}_{0}$$

The state vector consists of the suspension deflection $\left(z_{1}-z_{2}\right)$, the absolute velocity of the sprung mass ${\dot{z}}_{1}$, tire deflection $\left(z_{2}-z_{0}\right)$, and absolute velocity of the unsprung mass ${\dot{z}}_{2}$.

### 2.2. Active Aerodynamic Control Surface

**Figure 2:**
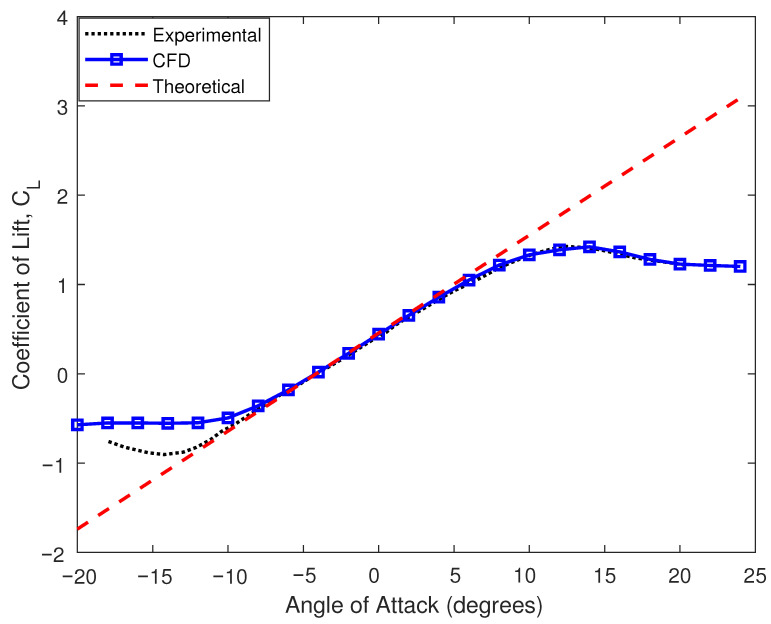
The generation of lift force using the AAS.

**Figure 3:**
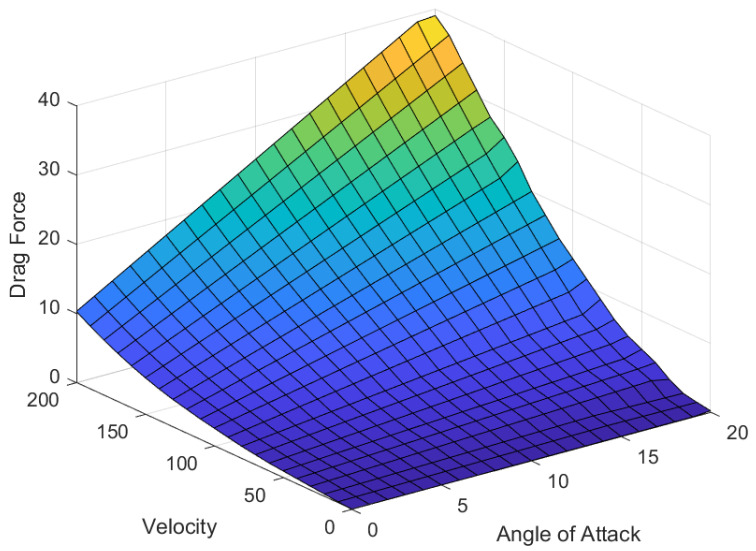
The generation of drag force using the AAS.

During vehicle’s translatory motion, it is exposed to an air pressure gradient that tends to exert an upward lift or downward force in the vertical direction on the vehicle’s chassis. Similarly, the drag force acts in the horizontal direction against the vehicle’s longitudinal motion. From the detailed investigation of the wind tunnel and CFD analyses carried out in [19], it is found that the aerodynamic drag coefficient $C_{D}$ depends on the angle of attack, geometry, surface conditions, and thickness of the AAS. Figure 2 and Figure 3 show the magnitude of these forces and illustrate their dependencies on the changing velocity and angle of attack. The characteristics of movable aerodynamic elements enhance driving safety, reduce carbon emissions, and improve traction and braking capabilities during wind gusts and moving obstacles [20]. The airfoils can increase the aerodynamic efficiency by increasing the lift-to-drag ratio at high angles of attack [21]. The control surface can be integrated with a conventional electronic control unit, enabling the vehicle to traverse a random road profile with added command and control capabilities. Thus, the manipulation of the vertical lift force changes the vertical dynamics of the vehicle. Equations (4) and (5) give the magnitude of these forces acting on the car body.

(4) $$F_{L}=\frac{1}{2}\rho_{air}v^{2}SC_{L}\left(\alpha \right)$$

(5) $$F_{D}=\frac{1}{2}\rho_{air}v^{2}SC_{D}\left(\alpha \right)$$

where $\rho_{air}$ is the air density, $C_{L}$ is the lift coefficient of the airfoil, *S* is the reference frontal surface area (m^2^) exposed to the air pressure, and α is the angle of attack. To study the effect of the aerodynamic force on the vertical dynamics of the suspension system, the study mainly focuses on the downward forces acting on the car body.

### 2.3. Road Preview Information

**Figure 4:**
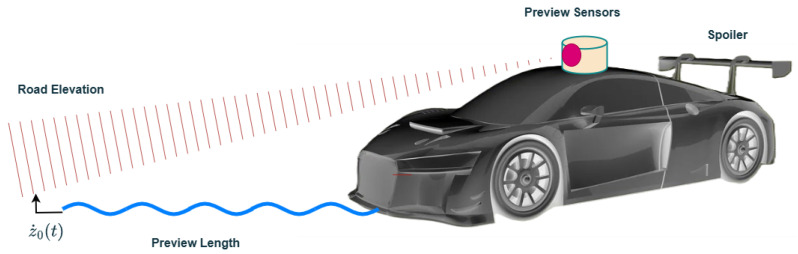
Car model with preview sensors.

To improve dynamic stability and trajectory planning, the wheel has to accurately track the road profile. A preview-based control algorithm with look-ahead capability has been reported in the literature [22,23,24]. To mitigate different road-induced dynamic excitations, the vehicle is assisted by in-camera or LiDAR-based optical road scanner sensors with road preview capability [25]. As show in Figure 4, the controller has future road information of the road input profile w(τ), over the interval $\tau \in [t,t+t_{p}]$, from the present time *t* to $t_{p}$ ahead of the vehicle. Usually, the preview time is less than the total duration, i.e., $t_{p}<T$, and the expression for the preview time is calculated as follows:

(6) $$t_{p}=L/v_{0}$$

where $v_{0}\left(t\right)$ is the vehicle velocity in the forward direction and *L* is the preview length. For a more realistic situation, e.g., white Gaussian noise with a given standard deviation and a deterministic road bump velocity input, the onboard preview road sensors are often considered. As the distance from the sensor increases beyond 0.3 s, the preview-based sensor data saturates and has no effect on the performance of the system [26].

## 3. Problem Formulation

**Figure 5:**
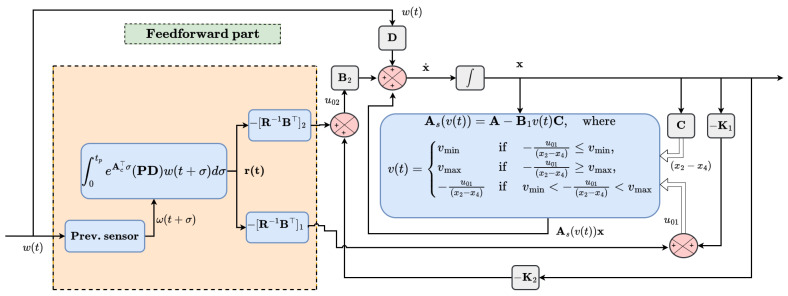
Block diagram of SASS with AAS showing optimal clipped algorithm.

The performance characteristics of the suspension system is a multi-objective problem that requires the analytical solution of the continuous-time algebraic riccati equation (ARE). The design of a vehicle suspension system involves conflicting objectives of ride comfort and handling characteristics. For passenger comfort, the variation in the passenger compartment’s vertical acceleration, which describes the discomfort level of the occupants, has to be kept smooth and minimal. Also, to increase the traction force on the ground surface, the tire deflection, which is proportional to the dynamic tire–road forces, must be kept small. A compromise has to be made between these objectives while maintaining the suspension travel requirement within the nominal value. As shown in Figure 5, an optimal clipped control algorithm is used to select the variable damper coefficient. For controller synthesis and efficient numerical simulation analysis, the following model simplifications are considered:

- The nonlinear dynamics of the actuator force and airfoil are neglected.
- For the given speed and road conditions, the horizontal drag force is neglected.
- Since our analysis is mainly related to the attenuation of external road-induced disturbances, the analysis will focus on the attenuation of these disturbances.

## 4. Design of Optimal Preview Controller

The optimal preview control of the SASS is typically represented in a conventional stochastic framework. However, to make the analysis easier and simpler, the model is expressed with perfect knowledge of the system state, as well as the road’s input bump elevation and random road excitations. For a given multi-objective optimization problem, the aim of this part is to design a preview-based optimal control system to minimize variation in the body acceleration and enhances wheel–road adhesion. To reconcile the tradeoff between passenger comfort and road holding under suspension travel and passivity constraints, different weighting factors with suitable values are optimized using trial-and-error method. Based on the previewed sensor information, the anticipated excitations for road irregularities are tracked 0.3 s earlier before they reach the system. Equation (7) shows the modified state equation of the model.

(7) $$\dot{x}=Ax-B_{1}v\left(t\right)\left(x_{2}-x_{4}\right)+B_{2}u_{2}+Dw\left(t\right)\;\;\;x\left(0\right)=x_{0}$$

At the instant of starting the simulation, the variable damper v(t) is assigned a zero value. The system matrix A and the constant matrices $B_{1}$, $B_{2}$ and D are expressed as follows:

(8) $$A=\left[\begin{array}{cccc} 0 & 1 & 0 & -1\\ -\frac{k_{1}}{m_{1}} & -\frac{b}{m_{1}} & 0 & \frac{b}{m_{1}}\\ 0 & 0 & 0 & 1\\ \frac{k_{1}}{m_{2}} & \frac{b}{m_{2}} & -\frac{k_{2}}{m_{2}} & -\frac{b}{m_{2}} \end{array}\right],B_{1}=\left[\begin{array}{c} 0\\ \frac{1}{m_{1}}\\ 0\\ -\frac{1}{m_{2}} \end{array}\right],B_{2}=\left[\begin{array}{c} 0\\ \frac{1}{m_{1}}\\ 0\\ 0 \end{array}\right],D=\left[\begin{array}{c} 0\\ 0\\ -1\\ 0 \end{array}\right],C={\left[\begin{array}{c} 0\\ 1\\ 0\\ -1 \end{array}\right]}^{⊤}$$

where $B=[B_{1},B_{2}]$. The determination of the variable damping coefficient for this nonlinear system requires the solution of the state-space equation of the fully active suspension system augmented by AAS under the preview-based optimal clipped control strategy. For robust performance, the system is optimized utilizing the following performance criterion:

(9) $$J_{act}=\lim_{T\to \infty }\frac{1}{2T}\int_{0}^{T}\left[{\ddot{z}}_{1}^{2}+\rho_{1}{\left(z_{1}-z_{2}\right)}^{2}+\rho_{2}{\left(z_{2}-z_{0}\right)}^{2}+\rho_{3}u_{01}^{2}+\rho_{4}u_{02}^{2}\right]dt$$

The weighting factors $\rho_{1}$ to $\rho_{4}$ are fine-tuned to optimize system performance, taking into account the influence of road irregularities and vehicle velocity. Alternatively, the performance criterion of this particular problem is expressed in matrix form as follows:

(10) $$J_{\mathrm{act}}=\lim_{T\to \infty }\frac{1}{2T}\int_{0}^{T}\left[x^{⊤}Qx+2x^{⊤}Nu_{0}+u_{0}^{⊤}Ru_{0}\right]dt$$

where $u_{0}={\left[u_{01},u_{02}\right]}^{⊤}$. For the SASS, the variable damper coefficient and the active aerodynamic force that minimize the quadratic cost function is expressed by replacing the variable damper control force as follows:

(11) $$\begin{array}{c} -v\left(t\right)\left(x_{2}-x_{4}\right)\Rightarrow u_{01} \end{array}$$

(12) $$\begin{array}{cc} u_{2} & =u_{02} \end{array}$$

In the best-case scenario, the performance of the variable damper coefficient approaches that of the active actuator force. Later in this paper, Equation (22) describes this condition. In accordance with the objective cost function, the constant weighting matrices for the state and control term are denoted by Q and R, while the cross-term weighting matrix is denoted by N. All matrices are given below.

(13) $$Q=\left[\begin{array}{cccc} \rho_{1}+\frac{k_{1}^{2}}{m_{1}^{2}} & \frac{k_{1}b}{m_{1}^{2}} & 0 & -\frac{k_{1}b}{m_{1}^{2}}\\ \frac{k_{1}b}{m_{1}^{2}} & \frac{b^{2}}{m_{1}^{2}} & 0 & -\frac{b^{2}}{m_{1}^{2}}\\ 0 & 0 & \rho_{2} & 0\\ -\frac{k_{1}b}{m_{1}^{2}} & -\frac{b^{2}}{m_{1}^{2}} & 0 & \frac{b^{2}}{m_{1}^{2}} \end{array}\right],R=\left[\begin{array}{cc} \rho_{3}+\frac{1}{m_{1}^{2}} & \frac{1}{m_{1}^{2}}\\ \frac{1}{m_{1}^{2}} & \rho_{4}+\frac{1}{m_{1}^{2}} \end{array}\right],N=\left[\begin{array}{cc} -\frac{k_{1}}{m_{1}^{2}} & -\frac{k_{1}}{m_{1}^{2}}\\ -\frac{b}{m_{1}^{2}} & -\frac{b}{m_{1}^{2}}\\ 0 & 0\\ \frac{b}{m_{1}^{2}} & \frac{b}{m_{1}^{2}} \end{array}\right]$$

The objective of this work is to effectively address the conflicting objectives of minimizing variations in the comfort-oriented index and ensuring a tight grip on the road surface for enhanced driving safety. Consider the following defined equation:

(14) $$A_{n}=A-{\mathrm{BR}}^{-1}N^{⊤}$$

(15) $$Q_{n}=Q-{\mathrm{NR}}^{-1}N^{⊤}$$

where $A_{n}$ is stable and $Q_{n}$ is a symmetric and non-negative matrix [27]. For the given problem, assuming that the pair $\left(A_{n},B\right)$ is stabilizable and the pair $\left(A_{n},Q_{n}^{1/2}\right)$ is detectable, then control law minimizing the suspension performance criterion can be stated as follows:

(16) $$u_{0}=-R^{-1}\left[\left(B^{⊤}P+N^{⊤}\right)x+B^{⊤}r\left(t\right)\right]$$

where $K={\left[K_{1},K_{2}\right]}^{⊤}=-R^{-1}\left(B^{⊤}P+N^{⊤}\right)$ for the active suspension system with an active aerodynamic element and preview information [28]. From the preview controller, the control law consists of a feedforward, $-R^{-1}B^{⊤}r\left(t\right)$, and feedback, $-R^{-1}\left(B^{⊤}P+N^{⊤}\right)$, term. The feedback term aims to minimize the deviation in the steady-state error, while the feedforward controller acts on the incoming road disturbance ahead of the vehicle. Depending on the control input, the aerodynamic control surface acting on the sprung mass either exerts lift or a downward force. The design of an optimal preview controller in conjunction with the AAS is realized by solving the ARE [29,30].

(17) $$A_{n}^{⊤}P+{\mathrm{PA}}_{n}-\mathrm{PB}R^{-1}B^{⊤}P+Q_{n}=0$$

where equation is solved for the positive definite symmetric P matrix. The control law used for calculating the variable damper coefficient of the SASS based on the optimal clipped strategy in comparison to the active actuator control force is depicted in Equation (18). Equation (19) gives the airfoil control force.

(18) $$u_{01}=-K_{1}x-{\left[R^{-1}B^{⊤}\right]}_{1}r$$

(19) $$u_{02}=-K_{2}x-{\left[R^{-1}B^{⊤}\right]}_{2}r$$

In the above two equations, subscript 1 depicts the first row of the actuator force accompanied by road preview information while subscript 2 elucidates the active airfoil control force in the control law vector. The preview vector, representing the numerical integration of the ground elevation within the preview horizon $\tau \in [0,t_{p}]$ in front of the vehicle, is defined as follows:

(20) $$r\left(t\right)=\int_{0}^{t_{p}}e^{A_{c}^{⊤}\sigma }\mathrm{PD}w\left(t+\sigma \right)d\sigma$$

The variable damping coefficient v(t) is capable of providing a limited damping force and switches between its maximum, minimum or no change values.

(21) $$v_{min}\leq v\left(t\right)\leq v_{max}$$

Equation (22) describes the control algorithm for the calculation of the variable damper coefficient [31]. Based on the selected value, the subsequent time-varying system matrix is updated in every iteration.

(22) $$\begin{array}{c} v\left(t\right)=\left\{\begin{array}{cc} v_{min} & \mathrm{if}\;-\frac{u_{01}}{\left(x_{2}-x_{4}\right)}\leq v_{min},\\ v_{max} & \mathrm{if}\;-\frac{u_{01}}{\left(x_{2}-x_{4}\right)}\geq v_{max},\\ -\frac{u_{01}}{\left(x_{2}-x_{4}\right)} & \mathrm{if}\;v_{min}<-\frac{u_{01}}{\left(x_{2}-x_{4}\right)}<v_{max}. \end{array}\right. \end{array}$$

From Equation (22), it is evident that the displacement vector $\left(x_{2}-x_{4}\right)$ can result in discontinuity when a singularity occurs. To avoid this condition, the preview controller preserves the previously calculated value in the control loop. To ensure optimal performance, the least square difference between the actuator force of the fully active suspension system and that of the SASS must be minimized under the given passivity constraints. The modified state Equation (23) utilizes the updated value of the variable damper coefficient.

(23) $$\begin{array}{c} \dot{x}=\left\{\begin{array}{cc} A_{s}\left(v_{min}\right)x+B_{2}u_{2}+Dw, & \mathrm{if}\;-\frac{u_{01}}{\left(x_{2}-x_{4}\right)}\leq v_{min},\\ A_{s}\left(v_{max}\right)x+B_{2}u_{2}+Dw, & \mathrm{if}\;-\frac{u_{01}}{\left(x_{2}-x_{4}\right)}\geq v_{max},\\ A_{s}\left(v\left(t\right)\right)x+B_{2}u_{2}+Dw, & \mathrm{if}\;v_{min}<v\left(t\right)=-\frac{u_{01}}{\left(x_{2}-x_{4}\right)}<v_{max} \end{array}\right. \end{array}$$

Meanwhile, $A_{s}\left(v\left(t\right)\right)x=\mathrm{Ax}-B_{1}\left(v\left(t\right)\right)\mathrm{Cx}$. The closed-loop system matrix in the case of the SASS with look-ahead sensor information accompanied by the active airfoil force is illustrated in Equation (24).

(24) $$\dot{x}=A_{sc}x-B_{2}{\left[R^{-1}B^{⊤}\right]}_{2}r+Dw$$

where $A_{sc}=\left(A_{s}\left(v\left(t\right)\right)-B_{2}K_{2}\right)$.

## 5. Results and Discussion

The optimal quadratic regulator in conjunction with a preview controller supplemented by control AAS was realized in MATLAB^®^ (R2025b). A desktop computer with 12 Gen Intel^®^ Core^™^ i7-12700K, 2.10 GHz, and 32 GB RAM (Intel, Santa Clara, CA, USA) was employed for the computational analysis. Generally, humans are sensitive to heave excitations in the frequency range from 4 to 8 Hz [32], which corresponds to the dominant vibration modes affecting ride comfort. Therefore, minimizing the vertical acceleration within this range is crucial for enhancing passenger comfort. At the same time, the control algorithm enhances the tire grip on the road surface by leveraging the preview controller and the aerodynamics effect while satisfying the rattle space requirements. To assess the influence of control prioritization, simulations were carried out under different road input conditions. Correspondingly, the frequency domain analyses were performed to evaluate the influence of each frequency component on the suspension system’s dynamic response.

### 5.1. Frequency-Domain Numerical Simulation

The primary concerns in the frequency domain include passenger comfort, suspension deflection, and maintaining firm contact between the tire and the ground surface. Generally, the vibration spectrum from 0 to 25 Hz falls into the category of noise, while the range from 25 Hz to 20 kHz is classified as harsh signals [33]. For linear time-invariant systems, the response can be calculated by using the Fourier transform of the closed-loop transfer function. In the case of the SASS, the variable damper coefficient varies with time in every iteration. Hence, the frequency response for the SASS is generated by taking the amplitude ratio of the state and input harmonic signal at the steady state. Equation (25) describes the method used to generate the frequency response in the time domain.

(25) $$\chi =\frac{max(\left|X(t)\right|)}{A_{max}\left(w\left(t\right)\right)}$$

**Figure 6:**
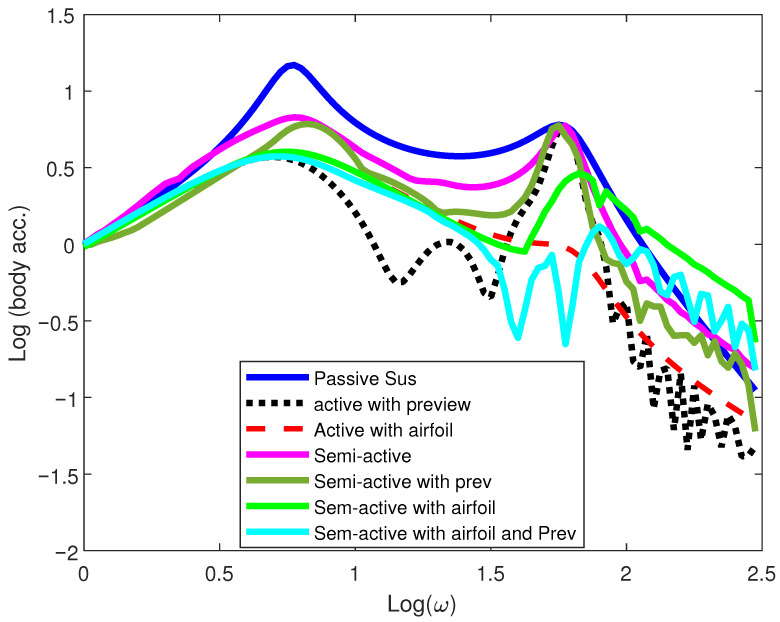
Passenger vertical acceleration response of 1/4-car model.

The results show that the response has two regions that influence the vertical dynamics. The body(chassis) dynamics are characterized by the bandwidth (1–5) Hz, while the wheel dynamics are concentrated in the range of (10–20) Hz [34]. These are the car body and the wheel resonance frequencies, which show invariant behavior regardless of the damping coefficient and spring stiffness. The system exhibits these invariant properties for passive, active, and semi-active suspension systems. On the other hand, load-leveling can regulate the static load of the vehicle but has no effect on the vehicle’s vertical dynamics. Figure 6 illustrates the frequency response characteristics of the vertical acceleration of various systems when the vehicle traverses a harmonic velocity input.

From the results, the SASS with a movable aerodynamic surface and preview information has the best response in terms of reducing both the resonant frequencies compared to the passive, semi-active, and active suspension with preview information. While the proposed model substantially improves the road holding metric at the wheel resonant frequency, the low-frequency response near the chassis resonant peak closely matches that of the active suspension system with preview information.

**Figure 7:**
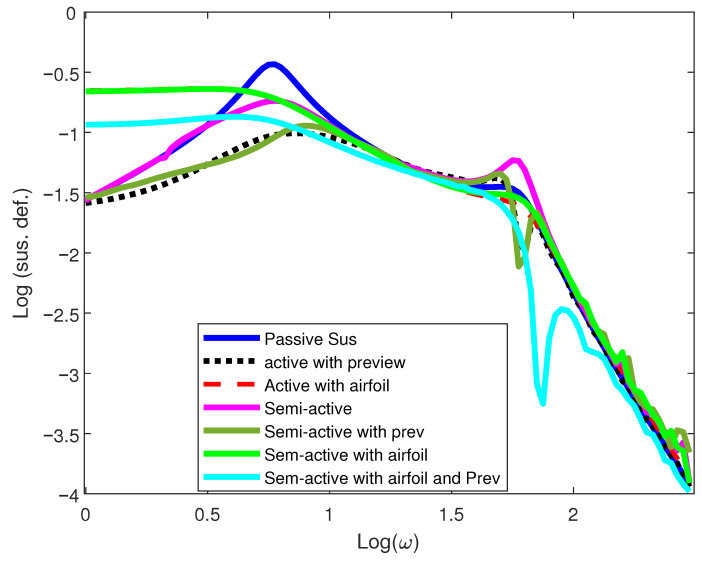
Suspension deflection response of 1/4-car model.

To preserve the dynamic suspension travel, the response must remain within the safe operating limits during the travel [35]. From the Figure 7, it can be observed that the suspension rattle space requirement at the first resonant peak compared to the active with airfoil and the PSS, is reduced. However, the low-frequency response is worse than that of a passive and semi-active suspension system. But it keeps on improving, compared to semi-active and active suspension with an active airfoil near the second hop frequency.

**Figure 8:**
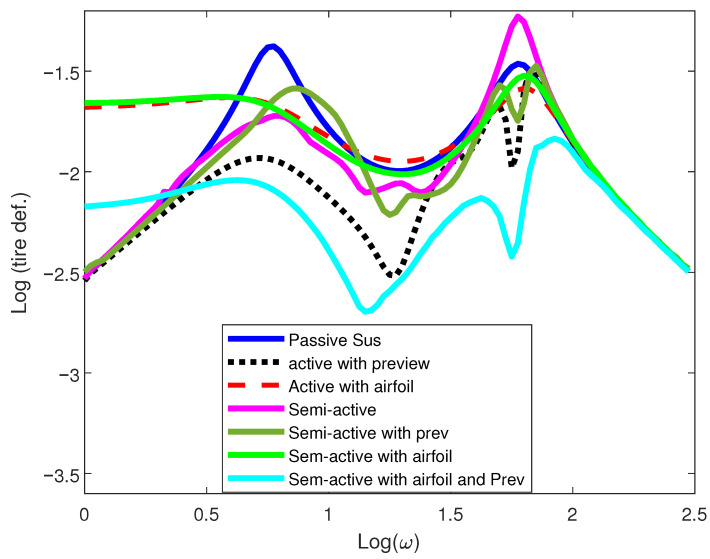
Road holding metric of 1/4-car model.

The aerodynamic element of the suspension system, in collaboration with the preview controller, significantly reduces tire hop frequency and enhances the tire grip on the road surface. Figure 8 depicts the dynamic tire loading characteristics of the vehicle. The preview controller typically performs well by anticipating the road kinematic excitation ahead of time, resulting in improved tracking and traction ability of the vehicle. The overall frequency analysis indicates noticeable improvement in the target indices of the model.

### 5.2. Time-Domain Numerical Simulation

In this section, detailed comparative analyses are presented for passive, active with preview, active with an active airfoil, semi-active with preview controller, and SASS with active aerodynamic surfaces. The International Organization for Standardization (ISO) published the standard ISO 2631-1:1997, which quantifies the impact of external disturbances on passenger comfort [36,37].

As a rule of thumb, the rms values of the comfort level lie in the range of $0.653m/s^{2}$ to $0.793m/s^{2}$ [38]. The computation analyses were performed both for deterministic bump velocity input and random road profile [39]. The bump input is manifested in the form of transient excitation, which led to passenger discomfort. Therefore, the primary and key consideration is to emphasize passenger comfort by effectively attenuating it.

**Figure 9:**
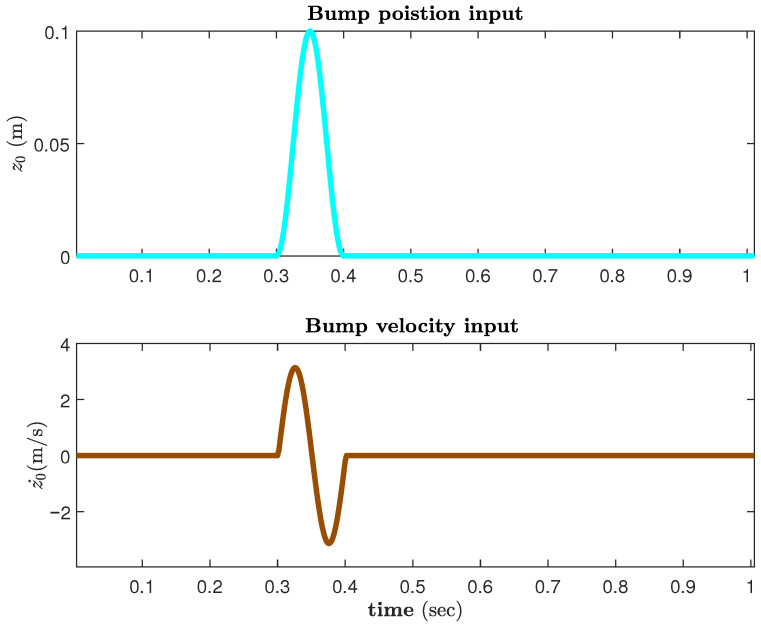
Bump position and input road velocity disturbance.

**Figure 10:**
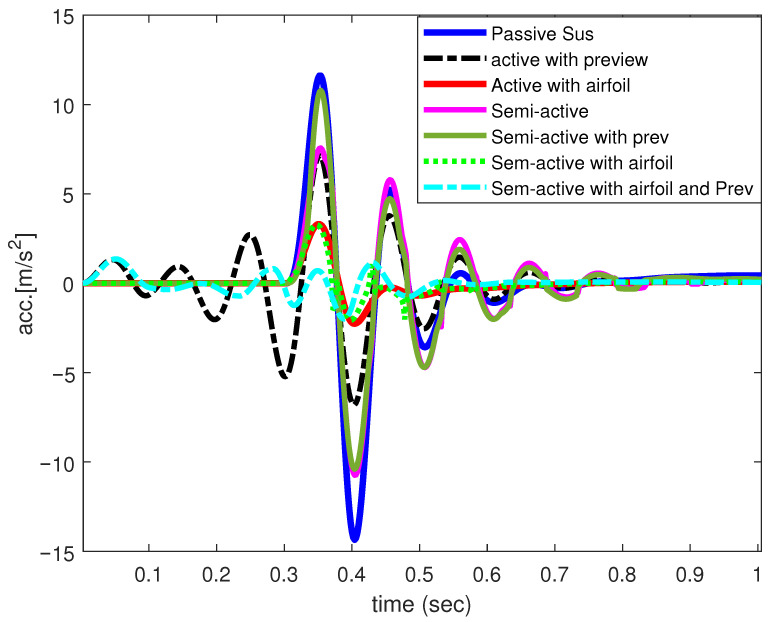
Vertical body acceleration of 1/4-car model.

**Figure 11:**
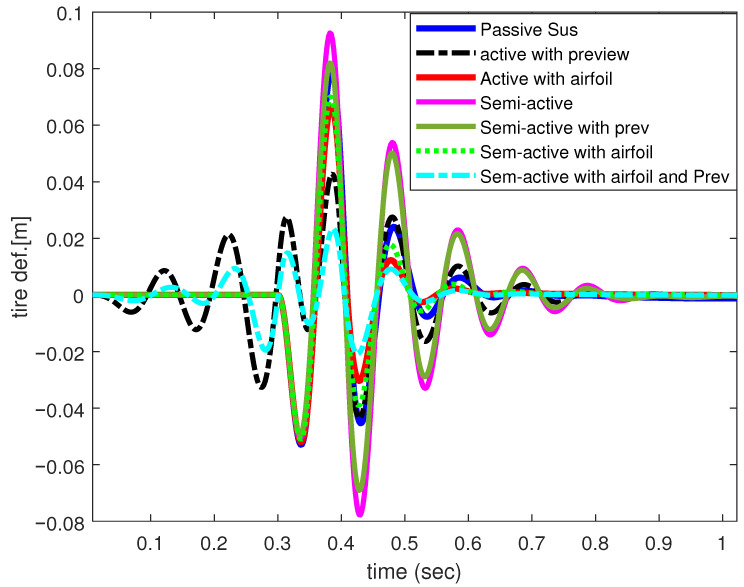
Tire deflection of 1/4-car model.

Figure 9 shows the input velocity for a position bump input of 0.1 m height. Figure 10 and Figure 11 illustrate the performance results achieved for the vertical body acceleration and road holding metrics. Table 2 presents the numerical results. For the given set of weighting factors the SASS with an active airfoil and preview controller, a significant improvement of 92.73% and 82.91% was achieved in the total cost function in comparison with passive and active suspension with airfoil. The passenger compartment acceleration saw an enhancement of 97.45% and 92.86%, respectively. At the same time, the road holding measure was improved by more than 80%.

**Figure 12:**
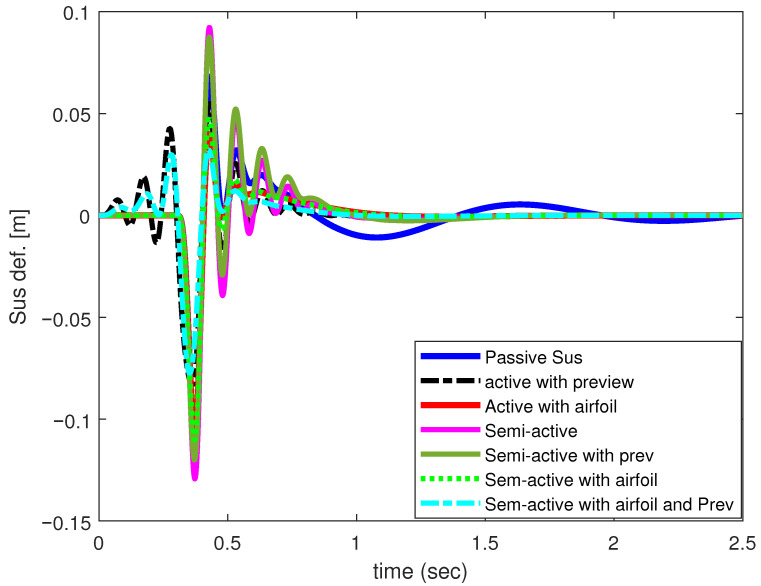
Suspension deflection of 1/4-car model.

From Figure 12, the response highlights that the suspension travel requirement preserves the limits and performs better than the corresponding PSS and active suspension with preview. Overall, the comparative results indicate marked improvement achieved by the proposed system.

The performance of the car model was evaluated for a random road profile. The white Gaussian noise disturbance velocity signal with zero mean and $\sigma^{2}=9\times 10^{-6}\;m^{2}$ variance excites the model. The car is assumed to travel with 20 m/s, and the roughness factor of the road surface is $0.15\;m^{-1}$. Table 3 displays the results for the random road profile. The displayed results indicate better results for both the car body acceleration and road grasping. The total performance measures remained at 86.41% and 63.96% compared to the passive system and active suspension with preview, while the passenger comfort markedly improved with percentage values of 92.93% and 77%, respectively. Meanwhile, the ground holding index remained greater than 70%. Compared to the two systems, suspension travel remained within the allowable range, corresponding to 65% and 43%, respectively. Overall, it can be concluded that the proposed design effectively mitigated the effect of external stimuli and enhanced the vertical performance and handling stability of the model.

## 6. Conclusions

In this work, the analytical formulation and numerical analyses of a SASS augmented by an active airfoil were successfully carried out through the application of an optimal preview control strategy. Both time- and frequency-domain numerical analyses were performed for the model validation. Initially, a comparative study for several vehicle suspensions was conducted to evaluate the frequency response analysis using the amplitude ratio of the transfer function. The SASS with an active airfoil and preview controller yields the best results in terms of reducing the invariant peaks of the car body and tire hop resonant frequencies. However, in the absence of active control force and and due to the passivity constraints, the SASS has a limited impact on enhancing the suspension travel and tire holding metrics prior to the first hop frequency. Overall, the impact of external stimuli on the road holding and suspension rattle space is considerably reduced, which shows marked improvement compared to other systems.

A comparative analysis of the different systems, for the time domain, was conducted for various road disturbances. From the numerical results, it is evident that the synthesized model improves the total performance criterion, vertical suspension acceleration, tire deflection, and suspension travel. To evaluate the system performance, two different road input disturbance scenarios were considered. In the first case, a discrete event like a road bump input signal shows 92% and 82% improvement over the passive and active with preview systems, respectively. The ride-oriented comfort exhibits improvements of 97% and 92%, respectively, and accompanied by substantial improvement in the tire road holding index. Also, better results were obtained when considering the impact of a random noise signal on the vehicle model. The proposed model outperforms all other systems in terms of passenger comfort, safety, and road gripping. To study the attitude motion, in addition to ride comfort and road holding, half- and full-car models will be used in the future.

## Figures and Tables

**Table 1:** Quarter-car suspension system model parameters.

Definition	Parameter	Value	Unit
Sprung mass	$m_{1}$	$m_{1}=1$	kg
Unsprung mass	$m_{2}$	$m_{2}=0.1\;m_{1}$	kg
Suspension stiffness	$k_{1}$	$k_{1}=36\;m_{1}$	N/m
Tire stiffness	$k_{2}$	$k_{2}=360\;m_{1}$	N/m
Passive damping coeff.	*b*	$b=0.5\;m_{1}$	N·s/m
Minimum damping coeff.	$v_{min}$	$v_{min}=0.5\;m_{1}$	N·s/m
Max. damping coeff.	$v_{max}$	$v_{max}=17\;m_{1}$	N·s/m

**Table 2:** The set of weighting factors ($\rho_{1}=10^{3},\;\rho_{2}=10^{4},\;\rho_{3}=0.1,\;\rho_{4}=0.1$).

Type	Body acc. (%)	Tire def. (%)	Sus. def. (%)	Cost (%)
Passive sus. sys.	100	100	100	100
Active sus. with preview	40.49	61.66	82.63	46.90
Active sus. with an airfoil	5.07	68.32	70.51	21.29
Semi-active suspension sys.	63.17	187.22	168.67	92.41
Semi-active sus. with preview	68.81	151.69	130.95	88.91
Semi-active sus. with AAS.	4.43	77.50	77.29	23.03
SASS with AAS and preview	2.55	13.93	51.21	7.27

**Table 3:** Weighting factors for random asphalt road disturbance signal ($\rho_{1}=10^{3}$, $\rho_{2}=10^{4}$, $\rho_{3}=0.1$, and $\rho_{4}=0.1$).

Type	Body acc. (%)	Tire def. (%)	Sus. def. (%)	Cost (%)
Passive sus. sys.	100	100	100	100
Active sus. with preview	35.62	67.37	53.96	43.71
Active sus. with an airfoil	7.71	75.53	69.63	27.04
Semi-active suspension sys.	57.45	154.73	101.60	81.17
Semi-active sus. with preview	54.50	88.54	66.79	62.52
Semi-active sus. with AAS.	8.07	84	72.52	29.28
SASS with AAS and preview	7.07	27.19	34.24	13.59

## Data Availability

All data are contained within the article.

## Abbreviations

The following abbreviations are used in this manuscript:

AAS	Active Aerodynamic Surface
SASS	Semi-active Suspension System
PSS	Passive Suspension System
ASS	Active Suspension System
MPC	Model Predictive Controller
KWLR	Knee Wheeled Legged Robot
V2V	Vehicle to Vehicle
LiDAR	Light Detection and Ranging
SMC	Sliding Mode Controller
QoV	Quarter of Vehicle
AV	Autnomous Vehicle
ER	Electrical–Rheological
DBC	Differential Braking Control
AAC	Active Aerodynamic Control
ARE	Algebraic Riccati Equation
